# H5N1 Whole-Virus Vaccine Induces Neutralizing Antibodies in Humans Which Are Protective in a Mouse Passive Transfer Model

**DOI:** 10.1371/journal.pone.0023791

**Published:** 2011-08-18

**Authors:** M. Keith Howard, Nicolas Sabarth, Helga Savidis-Dacho, Daniel Portsmouth, Otfried Kistner, Thomas R. Kreil, Hartmut J. Ehrlich, P. Noel Barrett

**Affiliations:** 1 Vaccine Research and Development, Baxter BioScience, Orth/Donau, Austria; 2 Global Research and Development, Baxter BioScience, Vienna, Austria; University of Texas at Tyler, United States of America

## Abstract

**Background:**

Vero cell culture-derived whole-virus H5N1 vaccines have been extensively tested in clinical trials and consistently demonstrated to be safe and immunogenic; however, clinical efficacy is difficult to evaluate in the absence of wide-spread human disease. A lethal mouse model has been utilized which allows investigation of the protective efficacy of active vaccination or passive transfer of vaccine induced sera following lethal H5N1 challenge.

**Methods:**

We used passive transfer of immune sera to investigate antibody-mediated protection elicited by a Vero cell-derived, non-adjuvanted inactivated whole-virus H5N1 vaccine. Mice were injected intravenously with H5N1 vaccine-induced rodent or human immune sera and subsequently challenged with a lethal dose of wild-type H5N1 virus.

**Results:**

Passive transfer of H5N1 vaccine-induced mouse, guinea pig and human immune sera provided dose-dependent protection of recipient mice against lethal challenge with wild-type H5N1 virus. Protective dose fifty values for serum H5N1 neutralizing antibody titers were calculated to be ≤1∶11 for all immune sera, independently of source species.

**Conclusions:**

These data underpin the confidence that the Vero cell culture-derived, whole-virus H5N1 vaccine will be effective in a pandemic situation and support the use of neutralizing serum antibody titers as a correlate of protection for H5N1 vaccines.

## Introduction

Highly pathogenic avian influenza (HPAI) viruses of subtype A/H5N1 continue to circulate in poultry and wild birds throughout Asia and Africa, causing sporadic human infections with a high case fatality rate. To date, at least 534 laboratory-confirmed human cases of H5N1 infections in 15 different countries have been recorded, leading to 316 confirmed deaths [1]. If H5N1 viruses gain the ability to transmit efficiently between humans, they have the potential to cause pandemics associated with significant human morbidity and mortality.

As part of pandemic preparedness strategies, vaccines against H5N1 and other HPAI viruses with pandemic potential are being developed. Timely evaluation of candidate pandemic vaccines will enable manufacturers and regulatory authorities to answer critical questions regarding safety, immunogenicity and efficacy in advance of large-scale immunization programs. A number of H5N1 vaccines have been shown to be safe and immunogenic in clinical trials and to protect rodents and ferrets from lethal challenge with wild-type viruses (reviewed in [2]).

We have developed a Vero cell culture platform which is being used for the large-scale production of both seasonal and pandemic influenza vaccines [3], [4]. Using the Vero platform, whole, inactivated pandemic vaccines derived both from clade 1 H5N1 A/Vietnam/1203/2004 and clade 2.1 A/Indonesia/05/2005 wild-type H5N1 virus strains have been developed. These vaccines have been shown to protect immunized mice from lethal challenge with both homologous and heterologous wild-type H5N1 viruses [5], [6]. Several clinical trials have also been undertaken in which the safety and potent immunogenicity of these vaccines has been consistently demonstrated [7]–[9]. In a phase I/II trial, 76% of subjects vaccinated with a non-adjuvanted 7.5 µg formulation developed neutralizing antibody titers of 1∶20 or more [8]. Compared with results from trials of non-adjuvanted split or subunit vaccines in which doses of 30 to 90 µg HA were required to induce adequate immune responses [10], [11], the whole-virus vaccine has significant dose-sparing potential, which may be critical in a pandemic scenario [12].

Cell culture-derived influenza vaccines also have several other potential advantages when compared to conventional egg-derived vaccines. Conventional methods for manufacturing influenza vaccines using embryonated chicken eggs are cumbersome, especially for highly pathogenic viruses such as H5N1 which require the generation of reassortant viruses. In contrast, Vero cells can be grown in modern, large-scale bioreactors, upscaling of vaccine production can be rapidly and consistently achieved, and all infectious production steps can be conducted at biosafety level 3, allowing the production of vaccines from highly pathogenic wild-type strains [7]. Moreover, the growth of influenza in eggs has been associated with the selection of antigenic variants that may be suboptimal for inducing protective antibodies to wild-type virus circulating in humans [13]–[15], whereas growth exclusively in mammalian-derived tissue culture was reported to be representative of the natural virus [16]–[18].

H5N1 infections are severely pathogenic in humans, but, since such viruses have yet to achieve efficient inter-human transmission, disease is not widespread and it is therefore difficult to determine clinical vaccine efficacy. Licensing guidelines for pandemic influenza vaccines have been developed via bridging to those established for seasonal influenza vaccines [2]. A better understanding of the relationship between the human antibody response elicited following immunization and protection from disease will facilitate the development of effective H5N1 vaccines.

The role of antibodies in protection from disease can be investigated using passive transfer of immune sera to animal models followed by challenge with lethal doses of wild-type virus. Passive transfer of vaccine-induced immune sera has been used to study the mechanisms of antibody-mediated protection against several highly pathogenic viruses including Nipah Virus [19], Andes virus [20], Japanese Encephalitis virus [21], Chikungunya virus [22] and Enterovirus 71 [23]. Passive transfer has also been used to evaluate the protective efficacy of human gammaglobulin or human monoclonal antibodies against West Nile virus [24], [25], Ebola virus [26], [27], and Dengue Fever virus [28]. In addition, passive transfer of vaccine-induced immune sera between mice was used to demonstrate the protective efficacy of a licensed pandemic H1N1v vaccine [29], and several studies have revealed the potential of monoclonal antibodies to protect animals from lethal challenge with wild-type H5N1 virus [30]–[40]. To date, however, investigations into the efficacy of H5N1 vaccine-induced human immune sera to protect against lethal challenge with wild-type virus have not been reported.

In the present study, we have evaluated the possibility of utilizing passive transfer of H5N1 vaccine-induced immune sera to bridge the data gap between vaccine immunogenicity observed in humans and protection from disease observed in animal models. Our results demonstrate that a Vero cell-derived, inactivated whole-virus H5N1 vaccine elicits potent humoral immune responses which following passive transfer protect mice against lethal challenge with wild-type H5N1 virus, and that protection correlates with serum neutralizing antibody titers.

## Materials and Methods

### Ethics Statement

Human immune sera were obtained as part of a registered clinical trial (ClinicalTrials.gov Identifier: NCT00462215) from volunteers who agreed to and understood the clinical study procedures and provided written consent to participation in the study. All participants signed an informed consent form prior to study entry permitting retained blood samples to be used for immunological testing, provided that this is for the further development of the vaccine. Clinical studies were conducted in compliance with Good Clinical Practice guidelines and the provisions of the Declaration of Helsinki. Ethics approval for clinical studies was obtained from the ethics committees of all institutions that participated in the study. Ethics approval was obtained from the Ethics Committee of the Medical Department of the Vienna General Hospital for clinical studies at the University Clinic for Clinical Pharmacology, Vienna General Hospital, Vienna, Austria, and the Centre for Travel Medicine, Vienna, Austria. Ethics approval was obtained from the Ethics Committee of Lower Austria for studies at the clinic of Dr. Reinhard Lober, Wiener Neustadt, Niederösterreich, Austria. Ethics approval was obtained from the Ethics Committee of the city of Vienna for clinical studies at the Vaccination centre Impfzentrum Nord, Vienna, Austria, and Sozialmedizinisches Zentrum Süd, Kaiser Franz Josef Hospital, Vienna, Austria. Ethics approval was obtained from the the Ethics Committee of the Medical Association of Rheinland-Pfalz for clinical studies at the Hautklinik, Mainz University Clinic, Germany and the practice of Dr. Schmitt and Dr. Regner, Mainz, Germany. Ethics approval was obtained from the Ethics Committee of Berlin for clinical studies at the Charité Research Organisation GmbH, Berlin, Germany.

All animal experiments were reviewed by the Baxter Institutional Animal Care and Use Committee and approved by the Austrian regulatory authorities. All animal experiments were conducted in accordance with Austrian laws on animal experimentation and guidelines set out by the Association for Assessment and Accreditation of Laboratory Animal Care International (AAALAC) and the US Department of Health and Human Services Office of Laboratory Animal Welfare (OLAW). Animals were housed according to OLAW and AAALAC guidelines, in housing facilities accredited by the AAALAC. The permit number granted by the Lower Austrian provincial government for the animal experiments performed during these studies is TVG-25/043-2006.

### Virus and vaccine

Wild-type A/H5N1/Vietnam/1203/04 virus (CDC#2004706280), obtained from the Centers for Disease Control and Prevention (CDC, Atlanta, USA) was propagated in serum-free Vero cell cultures. The formalin and UV light double-inactivated whole-virus vaccine was manufactured in an enhanced BSL-3 facility and has been previously described [8].

### Immune sera

Pooled sera were used for the majority of experiments since mice and guinea pigs are too small to allow serum from individual animals to be used to passively immunize groups of mice. Pools of immune sera were obtained from CD1 mice or guinea pigs by cardiac puncture 3 weeks following two immunizations, 3 weeks apart with 3.75 µg of non-adjuvanted vaccine. Mouse and guinea pig H5N1 antibody titers were determined by virus microneutralization (MN) assay, hemagglutination inhibition (HI) assay and ELISA. Pre-immune sera were collected for use as negative controls.

Human immune sera were collected 3 weeks following two immunizations, 3 weeks apart with 7.5 µg of vaccine, as part of a phase III H5N1 vaccine safety and immunogenicity clinical trial (ClinicalTrials.gov Identifier: NCT00462215). This trial recruited 583 participants, 561 of whom received at least one vaccination. Blood was obtained by arm venipuncture. Serum was obtained by letting blood rest at room temperature for 30 mins to 6 hours, followed by separation of serum from whole blood by centrifugation for 10 mins at 1100 to 1300 x g at room temperature. Serum was then frozen at ≤−20°C. All individual human immune sera collected as part of this trial were tested by MN assay, single radial hemolysis (SRH) assay and HI assay, as requested by both European and US regulatory agencies. Of these, antisera from 25 individuals were chosen for pooling, based on MN titers in individual sera. Pools consisted of sera from between 2 and 12 individuals.

### Passive immunization

CD1 mice were chosen for passive immunization experiments since they are outbred, thus better reflecting the human genetic situation. For single-injection experiments, groups of seven or ten 6–8 week old female CD1 mice were intravenously injected via the tail vein with 200 µl of a range of dilutions of pooled, non-heat-inactivated immune sera collected from mice, guinea pigs or humans. Antisera were diluted in naïve serum derived from the homologous species. Dilution factors were calculated to achieve desired final concentrations of serum neutralizing antibodies based on an estimated total blood volume of 1.5 ml [41]. For experiments which used 3 successive immunizations, groups of five or ten 6–8 week old female CD1 mice were injected intraperitoneally with 400 µl of undiluted human serum since, in our experience, this route is more convenient and reproducible for larger volumes of serum. Mice were bled 22 hours following passive immunization and sera from individual mice in each group were pooled to determine circulating H5N1 neutralizing antibody titers by MN assay.

### Virus challenge

Mice were challenged intranasally with 10^4^ tissue culture infectious dose fifty (TCID_50_), corresponding to 133 lethal dose fifty (LD_50_) of H5N1 wild-type virus (A/Vietnam/1203/2004), 24 h post-passive transfer. Mice were monitored for a period of 14 days for disease signs and death as a result of H5N1 challenge. Animals that survived 14 days post challenge were considered protected.

### CPE-based MN assay

H5N1 neutralizing antibody titers in pooled immune sera were determined via a cytopathic effect (CPE)-based MN assay as previously described [8]. Briefly, serum samples were heat inactivated at 56°C for 30 mins, then serially diluted with cell culture medium in two-fold steps. Dilutions were mixed 1∶1 with wild-type A/Vietnam/1203/2004 virus (100 TCID_50_ per well), incubated for 1 h at RT and eight-fold replicates per dilution were transferred to a microtiter plate with a Vero cell monolayer (1×10^4^ cells per well). After 5–7 days incubation at 37°C, the cultures were inspected for CPE. The neutralizing titer, expressed as the reciprocal of antiserum dilution at which virus growth is 50% inhibited (i.e. where 50% of wells show no CPE), was calculated by the number of virus negative wells and the serum dilution.

### Determination of PD_50_ values for immune serum neutralizing antibodies

The protective dose fifty (PD_50_), that is, the titer of anti-H5N1 serum neutralizing antibodies required to protect 50% of the challenged animals from death following challenge with wild-type virus, was calculated using an in-house software program [42]. To allow the PD_50_ to be calculated when this was below the limit of detection of the MN assay, measured MN titers in each experiment were compared with corresponding calculated values based on the original MN titer of the immunizing serum. This comparison was then used as the basis of linear regression analysis to extrapolate low dose serum antibody titers from the corresponding calculated titer.

## Results

### Passive transfer of H5N1 vaccine-induced immune sera provides dose-dependent protection of CD1 mice against wild-type H5N1 virus challenge

To evaluate the protective efficacy of immune sera elicited by a Vero cell culture-derived, whole-virus H5N1 vaccine, CD1 mice were passively immunized and subsequently challenged with a severe lethal dose of wild-type H5N1 virus. Pooled immune sera used for passive transfer experiments were obtained from immunized CD1 mice or guinea pigs, and human immune sera were collected during a H5N1 phase III clinical trial. H5N1 neutralizing antibody titers of undiluted pooled immune sera used for passive transfer experiments were 1∶465 (mouse sera), 1∶1347 (guinea pig) and 1∶147 to 1∶830 (human). Groups of ten 6-8-week old, female CD1 mice were passively immunized with a range of dilutions of immune sera calculated to achieve the desired circulating serum neutralizing antibody titers based on a total blood volume of 1.5 ml in recipient mice. Twenty-four hours following passive transfer, passively immunized mice were challenged intranasally with 10^4^ TCID_50_, (corresponding to 133 LD_50_) of wild-type A/Vietnam/1203/2004 H5N1 virus.

**Figure 1:**
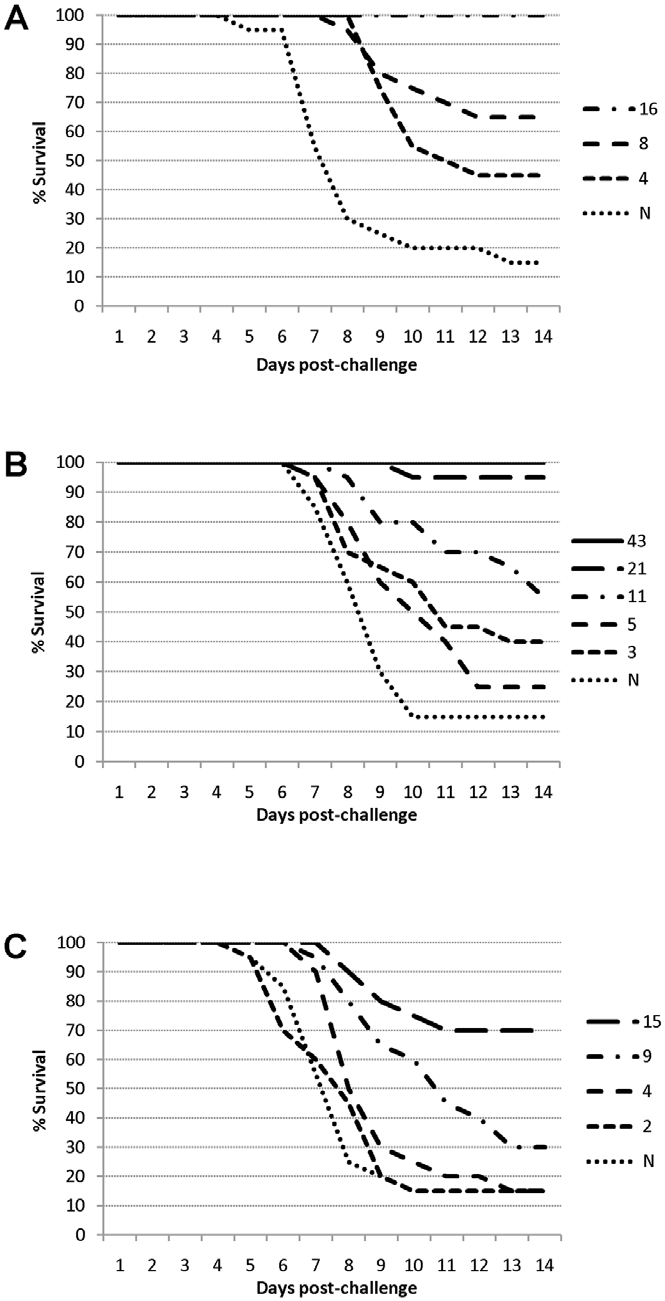
Prolongation of survival of passively immunized mice following challenge with wild-type H5N1 virus. Groups of 7 or 10 CD1 mice received immune sera from (**A**) CD1 mice, (**B**) guinea pigs or (**C**) humans and survival was monitored for 14 days. Shown are the mean % of surviving animals each day following lethal challenge. Reciprocal neutralizing antibody titers shown are the means extrapolated from titers measured immediately prior to challenge. N  =  Naive serum. All mice receiving mouse immune sera of mean titer ≥1∶16 and guinea pig immune sera of mean titer ≥ 1∶43 were protected.

Passive immunization provided dose-dependent protection against lethal H5N1 virus challenge. The data shown in Figure 1 indicate that even very low serum titers of neutralizing H5N1 antibodies were able to substantially delay the onset of death following lethal H5N1 challenge. More than half of passively immunized mice survived for at least 14 days following lethal challenge when circulating serum neutralizing antibody titers were calculated to be only 1∶8, 1∶11 and 1∶15 for mouse (Figure 1A), guinea pig (Figure 1B) and human (Figure 1C) whole-virus H5N1 vaccine-induced immune sera, respectively.

Complete protection of 100% of challenged mice for at least 14 days post-virus challenge was provided by mean serum H5N1 neutralizing antibody titers of 1∶16 or 1∶43 following passive transfer of mouse (Figure 1A) and guinea pig (Figure 1B) sera, respectively. Complete protection was also observed in all experiments where higher titers were used (Table 1). In initial experiments, complete protection was not provided by passive transfer of human immune sera at doses expected to achieve serum antibody titers of up to 1∶80. However, the maximum mean serum titer of H5N1 neutralizing antibodies detectable 22h after passive transfer of this dose of human immune sera was only 1∶29, representing a 64% reduction in titer. This rapid decrease in neutralizing antibody titer was only observed with human sera; neutralizing antibody titers measured in animals which were passively immunized with mouse or guinea pig sera were very similar to the calculated titers (Table 1). We next investigated whether full protection of mice might be attained by increasing the dose of human immune sera used for passive transfer. This was done by injecting mice with high titer human immune serum every day for three consecutive days, followed by lethal challenge with wild-type H5N1 virus 24 h after the third serum transfer. In four independent experiments, human immune sera calculated to result in circulating H5N1 antibody titers of between 1∶91 and 1∶159 were used to immunize a total of 35 mice. Actual serum neutralizing antibody titers of between 1∶22 and 1∶104 were measured 22 h following the third serum transfer. The increased serum neutralizing antibody titers achieved by injecting immune sera on three consecutive days were sufficient to protect 32 out of the total of 35 challenged mice, demonstrating that passive transfer of vaccine-induced human immune sera also has the potential to provide complete protection against H5N1 disease (Table 1).

**Table 1:** Dose-dependent protective efficacy of H5N1 vaccine-induced immune sera in mice.

	aExpected titer	bMeasured titer	cExtrapolated titer	dProtection; n/n (%)
**Mouse immune sera**	40	31	33	20/20 (100)
	20	19	16	20/20 (100)
	10	<9.4	8	13/20 (65)
	5	<9.4	4	9/20 (45)
	naive	<9.4	n.a.	3/20 (15)
**Guinea Pig immune sera**	160	170	171	20/20 (100)
	80	86	86	20/20 (100)
	40	45	43	20/20 (100)
	20	20	21	19/20 (95)
	10	11	11	11/20 (55)
	5	<9.4	5	5/20 (25)
	2.5	<9.4	3	8/20 (40)
	naive	<9.4	n.a.	3/20 (15)
**Human immune sera**	80	28	29	7/10 (70)
	50	17	15	20/30 (67)
	38	10	10	3/7 (43)
	30	8	8	11/30 (37)
	21	<7.1	6	7/20 (35)
	14	<7.1	4	7/40 (18)
	7	<7.1	2	6/40 (15)
	4	<7.1	1	6/20 (30)
	naive	<7.1	n.a.	6/40 (15)
				
	159^e^	104	n.a.	9/10 (90)
	140^e^	48	n.a.	9/10 (90)
	113^e^	31	n.a.	5/5 (100)
	91^e^	22	n.a.	9/10 (90)

Shown are reciprocal MN titers:

a expected titer based on the volume and titer of injected immune sera;

b measured circulating titer 2 h prior to challenge;

c extrapolated from titers measured 2 h prior to challengea.

d Mice were challenged intranasally with 10^4^ TCID_50_ wild-type H5N1 virus. Animals surviving for ≥14 days are considered protected.

e Immune serum administered on 3 successive days. n.a., not applicable.

### Serum neutralizing antibody titers correlate with protection from H5N1 disease

**Figure 2:**
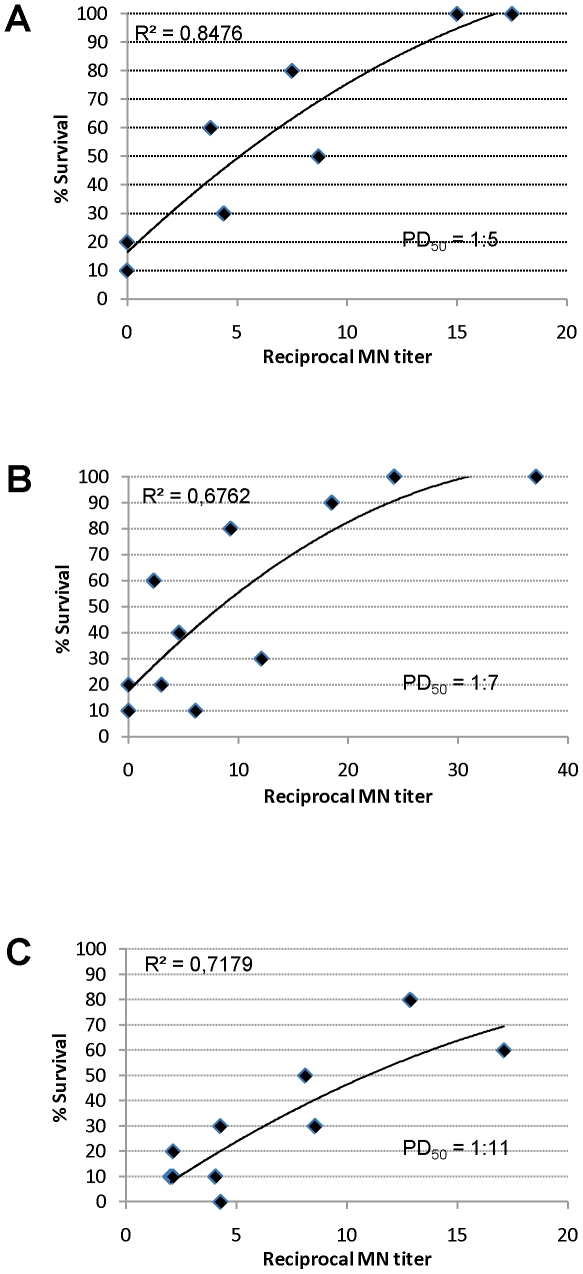
Correlation of survival of passively immunized mice with neutralizing antibody titer following challenge with wild-type H5N1 virus. Data points represent the mean % of surviving animals 14 days following lethal challenge of CD1 mice which had received immune sera from (**A**) CD1 mice, (**B**) guinea pigs or (**C**) humans. Reciprocal neutralizing antibody titers shown are extrapolated from titers measured 2 h prior to challenge. All individual mice receiving mouse immune sera of titer ≥1∶18 or guinea pig immune sera of titer ≥1∶37 were protected from disease; these animals are omitted from the figure to allow better resolution of lower titers.

Due to the lack of wide-spread H5N1 circulation, immune correlates of vaccine-induced protection have not been established for H5N1 vaccines. We therefore investigated the relationship between MN titer and death/survival in the mouse passive transfer model to determine whether MN titers could be used as an immune correlate of protection against H5N1 disease. The data in Figure 2 demonstrate that the titer of H5N1 whole-virus vaccine-induced serum neutralizing antibodies derived from immunized mice, guinea pigs and humans all correlated strongly with protection from severe H5N1 challenge upon passive transfer into mice.

The correlate of protection currently used for influenza vaccines is the vaccine-induced hemagglutinin (HA)-specific antibody titer, as measured by the HI assay. An HI titer of 1∶40, corresponding to prevention of disease in 50% of individuals, is used as a threshold for licensing purposes. To determine a threshold MN titer which corresponds to 50% protection against H5N1 disease, PD_50_ values were calculated for vaccine-induced mouse, guinea pig and human immune sera in the passive transfer model (Figure 2 A, B, C, respectively). The titer of serum neutralizing antibodies derived from all species correlated with protection from severe H5N1 challenge. The correlation co-efficient (r^2^) for mouse, guinea pig and human H5N1 immune sera was 0.85, 0.68 and 0.72, respectively. Moreover, PD_50_ values were similarly low regardless of serum source. Mouse, guinea pig or human immune sera were able to protect half of the challenged animals with neutralizing antibody titers of 1∶5, 1∶7 and 1∶11, respectively. These data indicate that a MN titer threshold of 1∶20 may be a conservative threshold appropriate for licensing purposes.

## Discussion

These studies were designed to investigate the ability of a Vero cell-derived, inactivated whole-virus H5N1 vaccine to induce antibodies which protect against highly pathogenic wild-type virus. This vaccine was previously demonstrated to protect immunized mice from challenge with lethal doses of wild-type virus [5], [6] and to be safe and immunogenic in human clinical trials [8], [9].

Due to the severity but current low incidence of human H5N1 disease, however, it is neither ethical nor feasible to demonstrate the clinical efficacy of H5N1 vaccines in placebo-controlled clinical trials. Here, we used a mouse passive transfer model to determine the protective efficacy of H5N1 vaccine-induced immune sera against wild-type H5N1 virus.

The data shown in Figure 1 demonstrate that immune sera from mice, guinea pigs or humans vaccinated with the whole-virus H5N1 vaccine provided dose-dependent protection following challenge with a severe lethal dose of wild-type H5N1 virus. Complete protection of recipient CD1 mice was provided by serum neutralizing antibody titers at or above 1∶16 and 1∶43 for mouse and guinea pig immune sera, respectively. However, a single transfer of human immune sera was insufficient to provide complete protection against virus challenge, probably due to the observed rapid decrease in human H5N1 neutralizing antibodies in the CD1 mouse model. However, complete protection could be achieved by repeated injections of human immune sera to increase circulating antibody titers.

This is the first report detailing the efficacy of immune sera elicited by vaccination of humans to protect against lethal H5N1 disease, thus it is not possible to make direct comparisons with other studies. However, a similar passive protection model in *C57BL/6* mice was used to demonstrate the potential of human immune sera elicited by an inactivated 2009 pandemic H1N1v vaccine to protect against challenge with the 1918 Spanish influenza virus [43]. In this study, mice passively immunized with 100 µl undiluted human immune sera survived for at least 14 days following challenge with 50 LD_50_ of 1918 virus, whereas all mice which received pre-vaccination sera died. We recently used a SCID mouse passive transfer model to evaluate the efficacy of antibody-mediated protection elicited by a Vero cell-derived, non-adjuvanted inactivated whole-virus 2009 pandemic H1N1v vaccine [29]. Passive transfer of 200 µl undiluted immune serum from CD1 mice or guinea pigs was found to protect 100% of SCID mice for at least 30 days, whereas all control animals died following challenge with a dose of 10^5^ TCID_50_ wild-type H1N1v virus.

Several groups have used mouse passive transfer models to demonstrate the protective efficacy of monoclonal antibodies against lethal H5N1 virus challenge [30]–[40]. It is difficult to compare the majority of such studies with the present work since neutralizing antibody titers are not commonly reported. One report [38] of passive protection studies using human monoclonal H5N1 antibodies from IgG^+^ memory B cells isolated from individuals who had recovered from H5N1 infection did however measure the neutralizing antibody titer of each antibody preparation against the same A/Vietnam/1203/2004 H5N1 virus used in the present study. From the data presented it can be calculated (assuming a recipient animal body weight of 20 g, total blood volume of 1.5 ml and 100% antibody recovery) that serum neutralizing antibody titers of approximately 1∶6 of the most potent monoclonal antibody protected 100% of Balb/c mice for at least 14 days following challenge with 10^5^ TCID_50_. A second monoclonal antibody clone derived from the same individual required titers of approximately 1∶60 to protect 80% of challenged mice. Thus, taking the different challenge doses into consideration, lower titers of some highly potent monoclonal H5N1 antibodies may provide full protection compared to vaccine-induced sera. This is to be expected since the polyclonal antibody response generated against vaccination with whole-virus H5N1 vaccine will consist of a mixture of high and lower potency neutralizing antibodies. However, whole-virus vaccine-induced polyclonal immune sera is likely to prevent the selection of escape mutants which may be associated with monoclonal antibodies [35].

A second goal of our mouse study described here was to determine the correlation between protective efficacy afforded by passive transfer of H5N1 vaccine-induced immune sera and associated H5N1 neutralizing antibody titers. Clinical correlates of vaccine-induced protection can only be calculated in human studies; however, since such studies cannot be carried out in the absence of widespread disease, passive transfer studies such as those reported here may provide a useful surrogate correlate of protection indicative of serum titers required to protect against clinical disease. Licensing guidelines for H5N1 vaccines are currently based on those established for seasonal influenza vaccines [2]. A reciprocal HI titer of 40 is generally accepted as being predictive of a 50% reduction in disease, and this threshold is used for licensing purposes [44]. However, the HI assay lacks sensitivity for the evaluation of H5N1 vaccines, and thus the more sensitive MN assay is commonly used for such studies [45]. In contrast to the HI assay, which only detects antibodies capable of preventing binding of influenza virus to erythrocytes, the MN assay detects all functional antibodies that interfere with infection. Hence, for H5N1 vaccines, MN titers may provide a better correlate of protection than HI titers. In the present study, MN titers were found to correlate strongly with protection from H5N1 disease (Figure 2). We thus used the MN assay to calculate the PD_50_ of serum neutralizing antibody titers associated with protection from lethal challenge with wild-type H5N1 virus. The titers of neutralizing serum antibodies required to protect 50% of animals from challenge with 10^4^ TCID_50_ (133 LD_50_) wild-type virus, regardless of serum source, was ≤1∶11 (Figure 2). A MN titer of 1∶20, as measured using the assay described here, has also been demonstrated to correlate well with a SRH seroprotective measurement of 25 mm^2^ in sera from human clinical trials [8].

Taken together, these findings support the confidence that the Vero-derived whole-virus H5N1 vaccine will be clinically protective in a pandemic situation. This conclusion is significant in that non-adjuvanted H5N1 vaccines have generally been reported to induce lower antibody titers than vaccines which include novel adjuvants [10], [11], [46], [47]. Also head-to-head studies with pandemic H1N1v vaccines have demonstrated that a novel adjuvanted H1N1v egg-derived vaccine induced significantly higher antibody titers than a non-adjuvanted Vero cell-derived H1N1v vaccine [48]. The studies reported here support the conclusion that lower neutralizing antibody titers are sufficient to confer protection, at least in a sensitive mouse model of lethal infection. These findings are also supported by the reports from an efficacy study with a seasonal influenza vaccine that demonstrated no additional protective efficacy was provided by HI antibody titers >1∶30 [49]. It also indicates that a MN titer of 1∶20 as determined by the assay described here could be used as a threshold for licensing purposes for H5N1 vaccines.
